# ﻿*Petrocosmea miechangensis* (Gesneriaceae), a new species from Yunnan, China

**DOI:** 10.3897/phytokeys.265.164730

**Published:** 2025-10-14

**Authors:** Sheng-Hu Tang, Cong-Rui Li

**Affiliations:** 1 Gesneriad Conservation Center of China (Guizhou), Guizhou Botanical Garden, Guiyang 550000, China; 2 National Forestry and Grassland Administration Key Laboratory for Biodiversity Conservation in Karst Terrain of Southwestern China, Guizhou Botanical Garden, Guiyang 550000, China; 3 Guizhou Academy of Forestry, Guiyang 550000, China

**Keywords:** Didymocarpoideae, flora of China, karst, Maguan County

## Abstract

A new species in Gesneriaceae, *Petrocosmea miechangensis*, is described from southeastern Yunnan, China. This species bears a resemblance to *P. sericea* in the shape of the adaxial corolla lip and in having very densely appressed hairs on the abaxial leaf blade. However, it can be distinguished by its flowering period, the hairs on the leaf blade, calyx, and filaments, as well as the shape of the filaments. An identification key to *P. miechangensis* and its related species is presented.

## ﻿Introduction

*Petrocosmea* Oliv. is a medium-sized genus within the family Gesneriaceae, subfamily Didymocarpoideae (Weber et al. 2013). The genus is indigenous to Asia and can be found in China, northeastern India, Myanmar, Thailand, Laos and Vietnam (Wang et al. 1998; POWO 2025). In 2015, the genus was divided into five major clades based on molecular evidence (Qiu and Liu 2015; Qiu et al. 2015). Yunnan Province is the center of *Petrocosmea* diversity (Wang 1985). In recent years, numerous new taxa have been discovered in Yunnan (IPNI 2025), including *P. rhombifolia* Y.H.Tan & H.B.Ding (Yang et al. 2019), *P. adenophora* Z.J.Huang & Z.B.Xin (Huang and Xin 2021), *P. purpureomaculata* M.Q.Han, J.Cai & J.D.Ya (Han et al. 2022), *P. hsiwenii* Lei Cai, J.D.Ya & J.Cai (Cai et al. 2022), *P. wangii* M.Q.Han, J.Cai & J.D.Ya (Han et al. 2024). As of June 2025, the genus *Petrocosmea* comprises 71 species and 4 varieties (GRC 2025).

In September 2022, Hui-Kui Wang, a plant enthusiast, collected some living plants of *Petrocosmea* with small leaf blades in Maguan County, Yunnan Province, China. Subsequently, he sold these plants online. We realized that these plants might represent a new species when they flowered in the Guizhou Botanical Garden in 2025. In May 2025, we collected specimens and took photographs in the wild with the assistance of Hui-Kui Wang. The plants produce flowers that closely resemble those of *P. martinii*, *P. sericea*, and *P. minor*, and they should be classified within sect. Minor Z.J.Qiu. The plants are distinguished by the small leaf blades, which are covered with densely appressed long hairs on the underside. After thorough comparisons, we concluded that they represented a new species.

## ﻿Materials and methods

The morphological characteristics of approximately 100 mature individuals were observed and 20 flowers selected were observed and measured carefully in the field. A microscope (Olympus SZ61, Tokyo, Japan) was used for micro-observation. The plant was described following the terminology used by Wang et al. (1998). The relevant literature comprised works by Hemsley (1899), Li (1983), Wang (1984), Wang et al. (1990),Wei and Wen (2009), Zhao and Shui (2010), Xu et al. (2011), Zhang et al. (2013), Qiu et al. (2015), Qiu and Liu (2015), Han et al. (2017, 2019),Wen (2019), Jiang et al. (2020), Li et al. (2020, 2023), Tang et al. (2021), Xu et al. (2022). Images of *Petrocosmea* type specimens were sourced from virtual herbaria and databases, including E (https://data.rbge.org.uk/search/herbarium/), K (http://apps.kew.org/herbcat/navigator.do), P (https://science.mnhn.fr/all/search), iPlant (http://www.iplant.cn/), and CVH (https://www.cvh.ac.cn/). Images of live *Petrocosmea* plants were sourced from iPlant (http://www.iplant.cn/). 22 species of *Petrocosmea* plants have been collected at the Guizhou Botanical Garden, including *P. cryptica*, *P. huanjiangensis*, *P. martini*, *P. sericea* and *P. xingyiensis*.

## ﻿Taxonomic treatment

### ﻿ Petrocosmea miechangensis

Taxon classificationPlantaeLamialesGesneriaceae

Sheng H.Tang & Cong R.Li sp. nov.

AFBB9223-E6D3-5F9D-9A3C-9614BD14B47C

urn:lsid:ipni.org:names:77370513-1

Figs 1, 2

#### Diagnosis.

The new species displays the following characteristics: the corolla tube is shorter than the limb, and the adaxial corolla lip is significantly shorter than the abaxial corolla lip. Consequently, it should be classified within sect. Minor Z.J.Qiu within the genus *Petrocosmea*. Species in sect. Minor Z.J.Qiu typically exhibit minor variations in their flowers, fruits and seeds, but they display significant differences in their leaves and hairs. *P. miechangensis* and *P. sericea* are found in Wenshan City, Yunnan Province, China and they both possess similar leaf blade hairs (the silvery-white, glossy, silk-like hairs are unique to the genus *Petrocosmea*). So *P. miechangensis* is most similar to *P. sericea* (Fig. 3). However, it diverges in several critical respects: it flowers from April to May, not in October, and the stamens and pistil form a triangular interspace (vs. a crescent-shaped one). Additionally, it features very densely appressed pilose (1.3–1.5 mm long) leaf blades on the underside (vs. densely appressed pubescent, 0.7–1 mm long), spreading pilose and glandular puberulent calyx lobes (vs. densely appressed pubescent), spreading pilose and glandular puberulent pedicels (vs. densely appressed sericeous pubescent), and filaments that are glandular puberulent and densely glandular pubescent (vs. densely puberulent), with a bend of roughly 90 degrees (vs. roughly 30 degrees).

**Figure 1.**
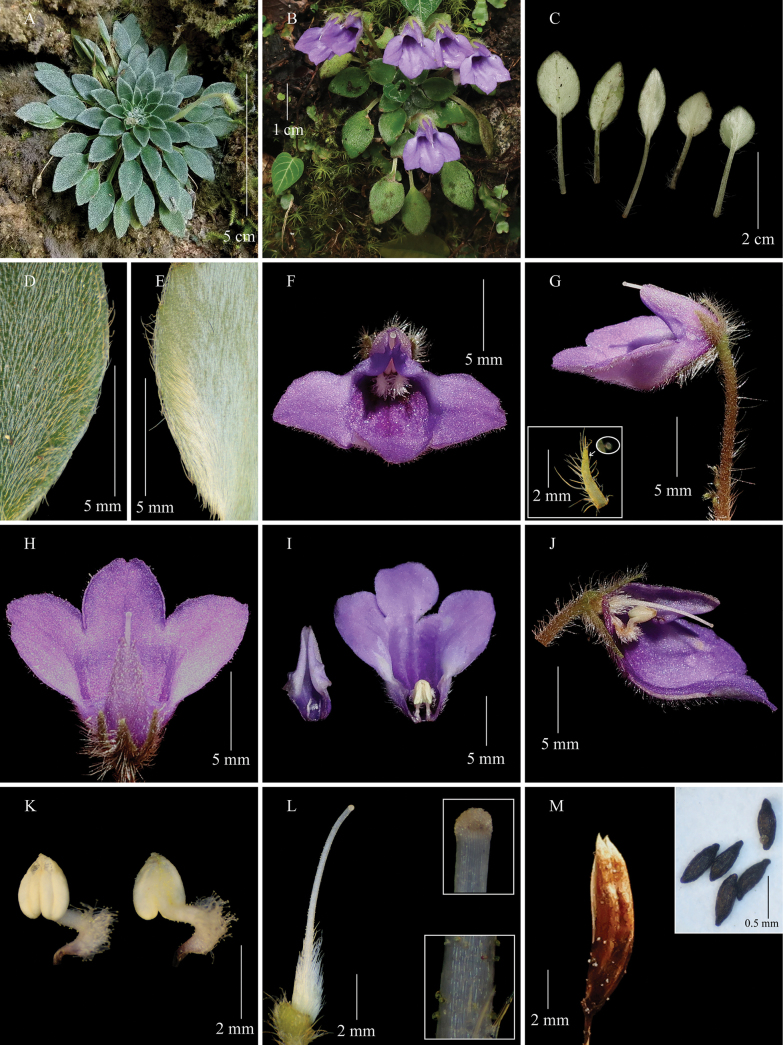
*Petrocosmea miechangensis* sp. nov. A. Habitat; B. Flowering plant; C, E. Abaxial surface of leaf blade; D. Adaxial surface of leaf blade; F. Flower in front view; G. Flower in side view and a calyx lobe (inset); H. Flower in top view; I, J. Opened corollas; K. Mature stamens; L. Pistil, stigma (inset, top) and a part of style (inset, down); M. Capsule and seeds (inset). (A was captured on camera by Hui-Kui Wang, while the others were photographed by Sheng-Hu Tang).

**Figure 2.**
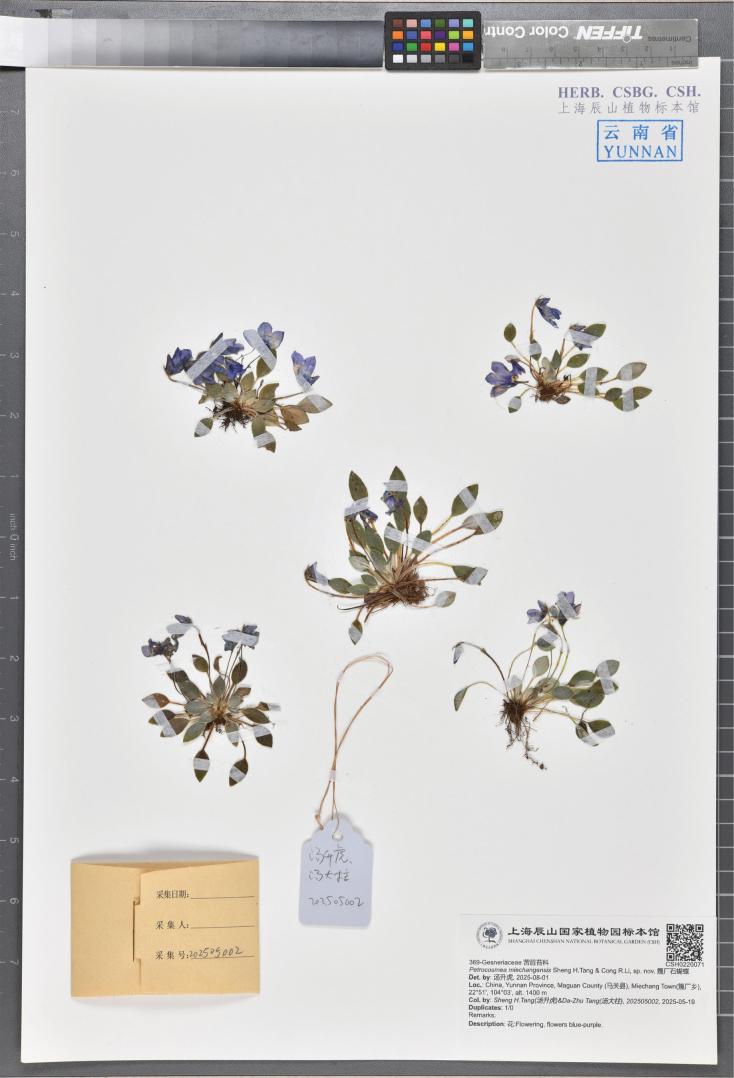
Holotype of *Petrocosmea miechangensis* sp. nov. stored in CSH (*Sheng H.Tang & Da-Zhu Tang 202505002*, CSH0220071).

**Figure 3.**
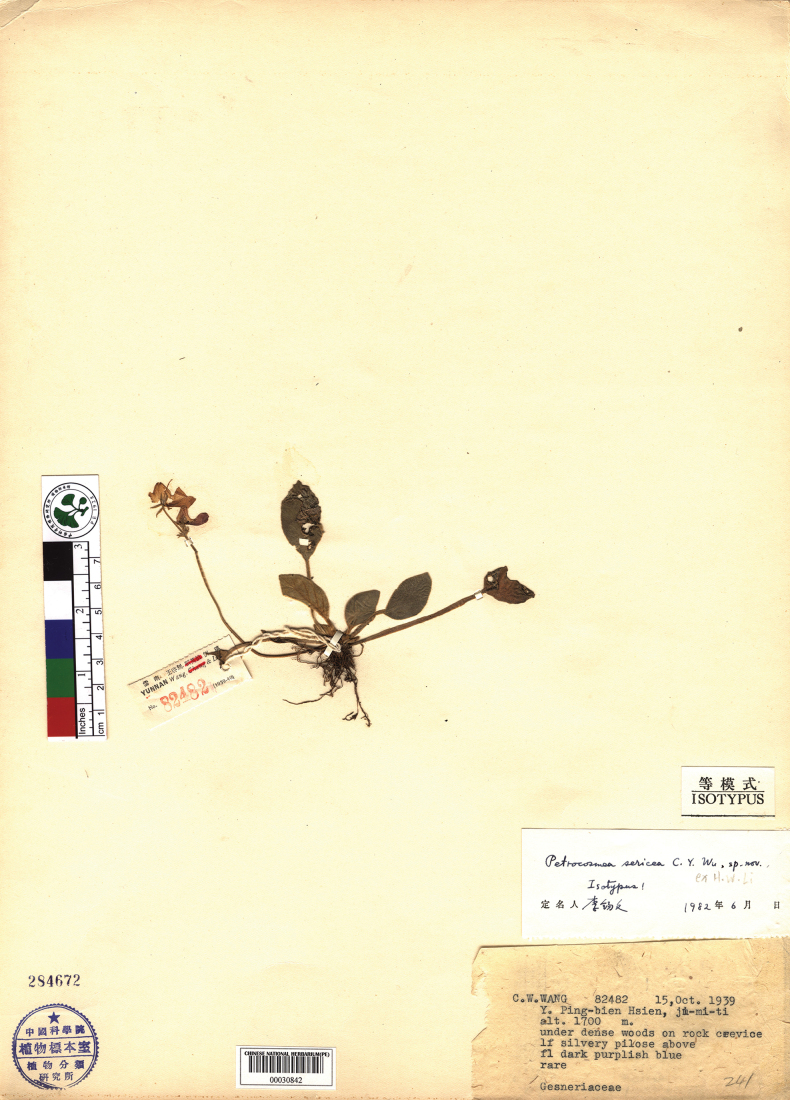
Isotype of *Petrocosmea sericea* stored in PE (*C.W.Wang 82482*, 00030842).

#### Type.

China • Yunnan Province: Maguan County, Miechang Town, 22°51'N, 104°03'E, ca. 1,400 m, 19 May 2025, *Sheng H.Tang & Da-Zhu Tang 202505002* (holotype: CSH! [accession number CSH0220071]; isotype: the Guizhou Botanical Garden!).

#### Description.

Perennial herbs. Rhizome short, 5–8 mm long, 4–5 mm in diameter. Leaves all in basal rosette, 15–60; inner leaves with petioles 0.5–7.5 mm long or absent, outer leaves with petioles 7.5–62 mm long, 0.8–1.3 mm in diameter, densely descending appressed pubescent and sparsely spreading villous; leaf blade papery when dry, ovate or elliptic, 8.2–20 × 4.3–12.5 mm, adaxially densely appressed pubescent, abaxially very densely appressed sericeous pilose (1.3–1.5 mm long), base cuneate or broadly cuneate, margin entire and sparsely spreading villous, sometimes shallowly crenate, apex acute or acuminate; lateral veins 2–3 on either side of midrib, adaxially and abaxially inconspicuous. Cymes 1–8, one flower per cyme; peduncle 14–24 mm long, 0.9–1.2 mm in diameter, spreading pilose and glandular puberulent; bracts 2 or 3, lanceolate, nearly equal, 1.6–2.9 × 0.3–0.5 mm, sparsely pubescent outside, glabrous inside; pedicels 8–16 mm long, spreading pilose and glandular puberulent. Calyx zygomorphic, spreading pilose and glandular puberulent outside, glabrous inside; adaxial calyx lip 3-parted nearly to base, abaxial calyx lip 2-parted to base, all segments nearly equal, lanceolate, 3.7–4.7 × 0.8–1 mm, margin entire, apex acuminate. Corolla blue-purple, 14.5–17 mm long, puberulent outside, glabrous inside; tube 3.8–4.7 mm long, 4.4–6 mm in diameter at mouth, 3–3.8 mm in diameter at base, broadly tubular, two dark blue-purple stripes inside tube beneath anthers; throat blue-purple, without stripes or with two inconspicuous stripes; limb distinctly 2-lipped, adaxial corolla lip 3.4–4.5 mm long, narrowly triangle, bent and keeled, apex emarginate, margin recurved, abaxial corolla lip 9.8–13.1 × 13.8–18.4 mm, broadly obovate, 3-parted to near middle, segments subequal, ovate, 4–6.7 × 5–7.5 mm. Stamens 2, connivent, adnate to corolla tube near base, included; filaments 2.6–3 mm long, with a bend of roughly 90 degrees, glandular puberulent, and adaxially densely glandular pubescent near middle; anthers coherent, ovate, 1.7–1.9 × 1.2–1.4 mm, basifixed, sparsely glandular puberulent on dorsal side; thecae two, parallel, not confluent, poricidal near apex. Staminodes three, included, adnate to corolla tube near base, 0.9–1.4 mm long, glabrous. Disc absent. Pistil 9–10.8 mm long; ovary densely appressed pubescent, narrowly ovoid or oblong, 1.1–2.2 mm long, 0.8–1.1 mm in diameter; style 7.9–8.6 mm long, sparsely glandular puberulent, and appressed pubescent near base; stigma nearly globose, ca. 0.3 mm in diameter, apex rounded. Capsule straight in relation to pedicel, 5–8 mm long, ca. 1.5 mm in diameter, oblong, glabrous, dehiscing loculicidally to base; valves 2, straight, not twisted. Seeds 0.4–0.6 mm long, ellipsoid, unappendaged.

#### Phenology.

Flowering occurs from April to May, fruiting in the wild is unknown. Only capsules of the previous year were observed.

#### Etymology.

The new taxon was named after its locality, Miechang Town, Maguan County, China.

#### Vernacular name.

The Chinese name is “Miè Chăng Shí Hú Dié” (篾厂石蝴蝶).

#### Distribution and habitat.

**Figure 4.**
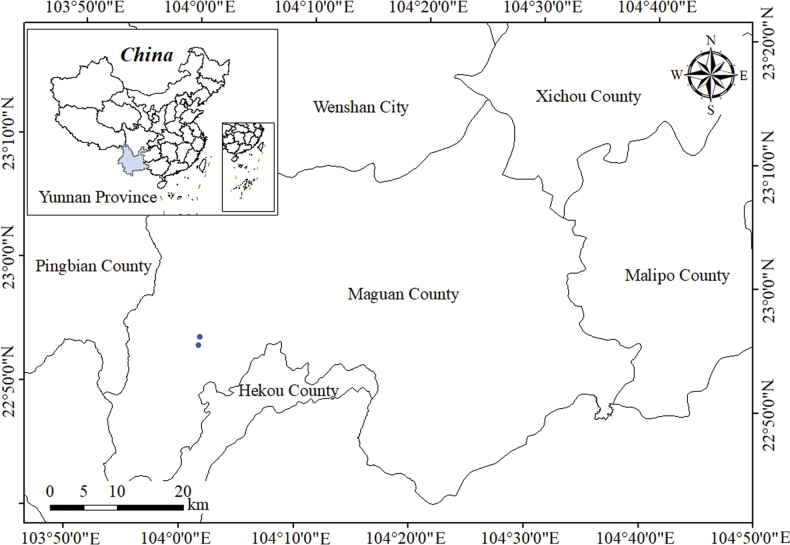
Locations of two populations of *Petrocosmea miechangensis* sp. nov. in Miechang Town, Maguan County, Yunnan Province, China (indicated by blue circles).

Two populations have been discovered in Miechang Town, Maguan County, China (Fig. 4). The plants thrive on moist, shady cliffs and limestone surfaces. The primary companion species are *Thalictrum ichangense* Lecoy. ex Oliv. (Ranunculaceae), *Oreocharis hekouensis* (Y.M. Shui & W.H. Chen) Mich.Möller & A.Weber (Gesneriaceae), *Anna mollifolia* (W. T. Wang) W. T. Wang & K. Y. Pan (Gesneriaceae), *Impatiens wenshanensis* S. H. Huang (Balsaminaceae), and *Clarkella nana* (Edgew.) Hook. f. (Rubiaceae).

#### Conservation status.

Two populations, totaling approximately 150 mature individuals, were discovered in Miechang Town, Maguan County, China. The straight-line distance between the populations is approximately 2 kilometers. Road construction could pose a potential threat to their survival. It is highly probable that additional populations exist in this region. Until further investigation is carried out, the species should be classified as “Data Deficient” (DD) following IUCN standards (IUCN 2024).

#### Taxonomic notes.

*Petrocosmea miechangensis* bears resemblance to 23 species and one variety of *Petrocosmea*, sharing the following characteristics: the corolla tube is shorter than the limb, the adaxial corolla lip is significantly shorter than the abaxial corolla lip, and the adaxial corolla lip apex is emarginate. An identification key for *P. miechangensis* and its related species is presented.

##### ﻿Key to *Petrocosmea miechangensis* and its related species

**Table:** 

1	Leaf blades base cuneate, broadly cuneate, rounded, or peltate	**2**
–	Leaf blades base cordate	**14**
2	Filaments straight	**3**
–	Filaments curved near middle	**5**
3	Filaments glabrous	***P. wangii***
–	Filaments densely glandular puberulent	**4**
4	Leaf blade adaxially densely ascending pilose	***P. chiwui***
–	Leaf blade adaxially glabrous	***P. rotundifolia***
5	Leaf blades base peltate	***P. huanjiangensis***
–	Leaf blades base not peltate	**6**
6	Corolla light green or white	**7**
–	Corolla blue or purple	**8**
7	Filaments densely puberulent	***P. cryptica***
–	Filaments glandular pilose	***P. viridis***
8	Abaxial leaf blades surfaces densely appressed hairs	**9**
–	Abaxial leaf blades surfaces densely erect or ascending hairs	**10**
9	Filaments densely puberulent, with a bend of roughly 30 degrees	***P. sericea***
–	Filaments glandular puberulent and densely glandular pubescent, with a bend of roughly 90 degrees	***P. miechangensis***
10	Leaf blades narrowly oblanceolate	***P. xingyiensis***
–	Leaf blades ovate, obovate or elliptic	**11**
11	Filaments villous	**P. shilinensis var. changhuensis**
–	Filaments glandular pubescent or glandular puberulent	**12**
12	Lateral lobes of abaxial corolla lip narrowly triangular	***P. getuheensis***
–	Lateral lobes of abaxial corolla lip ovate or broadly ovate	**13**
13	Filaments densely glandular pubescent	***P. minor***
–	Filaments densely glandular puberulent	***P. purpureoglandulosa***
14	Filaments glabrous, sparsely pilose, or densely pilose	**15**
–	Filaments densely glandular hairs	**18**
15	Anthers lanceolate	**P. shilinensis var. shilinensis**
–	Anthers ovate	**16**
16	Leaf blades 50–150 mm long	***P. funingensis***
–	Leaf blades 10–25 mm long	**17**
17	Filaments glabrous or sparsely pubescent	***P. leiandra***
–	Filaments densely pubescent	***P. qiruniae***
18	Leaf blades margins deeply lobed	***P. weiyigangii***
–	Leaf blades margins not deeply lobed	**19**
19	Style curved above base	**20**
–	Style straight	**21**
20	Corolla tube 4.6–5.4 mm long, apex of abaxial corolla lip lobes reflexed	***P. duyunensis***
–	Corolla tube 6–7 mm long, apex of abaxial corolla lip lobes not reflexed	***P. longituba***
21	Corolla glandular puberulent outside	***P. adenophora***
–	Corolla puberulent outside	**22**
22	Lateral lobes of abaxial corolla lip obovate or ovate	***P. dejiangensis***
–	Lateral lobes of abaxial corolla lip broadly ovate	**23**
23	Lateral veins adaxially conspicuous	***P. iodioides***
–	Lateral veins adaxially inconspicuous	**24**
24	Petioles densely erect pubescent	***P. qionglaiensis***
–	Petioles densely descending pubescent and sparsely spreading villous	***P. martinii***

## Supplementary Material

XML Treatment for Petrocosmea miechangensis

## ﻿Additional information

### Use of AI

No use of AI was reported.

### Author ORCIDs

Sheng-Hu Tang https://orcid.org/0000-0002-2168-8559

Cong-Rui Li https://orcid.org/0000-0003-3310-1660

### Data availability

All of the data that support the findings of this study are available in the main text.
